# Surface Modifications of Nanoparticles for Stability in Biological Fluids

**DOI:** 10.3390/ma11071154

**Published:** 2018-07-06

**Authors:** Luca Guerrini, Ramon A. Alvarez-Puebla, Nicolas Pazos-Perez

**Affiliations:** 1 Departamento de Quimica Fisica e Inorganica and EMaS, Universitat Rovira i Virgili Carrer de Marcel•lí Domingo s/n, 43007 Tarragona, Spain; luca.guerrini@urv.cat; 2 Institución Catalana de Investigación y Estudios Avanzados, Passeig Lluís Companys 23, 08010 Barcelona, Spain

**Keywords:** nanoparticles, biological fluids, colloidal stability, surface modification, protein corona, antifouling, plasmonics, quantum dots, magnetism

## Abstract

Due to the high surface: volume ratio and the extraordinary properties arising from the nanoscale (optical, electric, magnetic, etc.), nanoparticles (NPs) are excellent candidates for multiple applications. In this context, nanoscience is opening a wide range of modern technologies in biological and biomedical fields, among others. However, one of the main drawbacks that still delays its fast evolution and effectiveness is related to the behavior of nanomaterials in the presence of biological fluids. Unfortunately, biological fluids are characterized by high ionic strengths which usually induce NP aggregation. Besides this problem, the high content in biomacromolecules—such as lipids, sugars, nucleic acids and, especially, proteins—also affects NP stability and its viability for some applications due to, for example, the formation of the protein corona around the NPs. Here, we will review the most common strategies to achieve stable NPs dispersions in high ionic strength fluids and, also, antifouling strategies to avoid the protein adsorption.

## 1. Introduction

**Figure 1:**
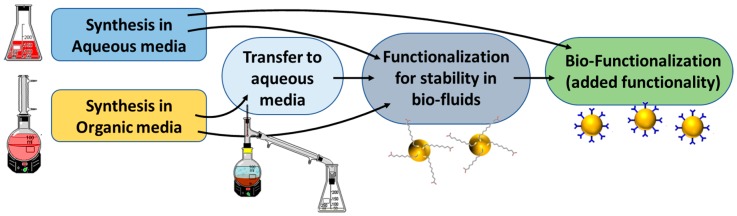
Strategies to achieve stable NPs in biofluids.

The use of inorganic nanoparticles (NPs) for biological and medical applications has attracted great attention in recent decades. This is clearly demonstrated by the large increase in publications reporting the use of nanotechnology for biomedical purposes [1,2,3,4,5,6,7,8,9,10], which can be summarized in three main properties arising from the nanoscale: (i) the similar size to biomacromolecules allows for a better interaction of NPs with cells and biomolecules (nucleic acids, proteins, lipid membranes, etc.) [11]; (ii) the high NP surface: volume ratio facilitates the incorporation of a high density of functional moieties [12]; and, (iii) the unique physicochemical properties (optical, electric, and magnetic) derived from the nanoscale size. The possibility of tailoring these properties on demand by modifying NP composition, size, and/or shape enables obtaining responsive materials toward very specific external electromagnetic stimuli [13,14]. All these features can be exploited in many potential applications such as MRI imaging [15], precise therapy at the cellular and subcellular levels with remotely triggered drug delivery (or hyperthermia) [12,16,17], early diagnostics of infectious diseases and cancer [18], detection of genetic mutations [19,20], or improving implant performance with antibacterial activity [21]. Therefore, the full understanding of the properties and behavior of NPs in biological media is critical. For conventional sols, the colloidal stability in such complex environments is usually compromised, resulting in aggregation and flocculation [22,23,24]. Broadly speaking, two main approaches are typically used for imparting colloidal stabilization. The most common and simplest strategy relies on the stabilization through electrostatic repulsion (e.g., citrate-stabilized NPs). At low ionic strengths, the diffuse double layer (DDL) extends far from the particle surface, facilitating particle–particle repulsions. However, at high ionic strengths, the NP DDL is compressed and neutralized with the subsequent aggregation due to van der Waals forces [25,26]. Thus, electrostatic stabilization largely fails to provide sufficient colloidal stability in biological media. The second method consists in the generation of a physical barrier at the NP surface (i.e., steric stabilization). For instance, polymers attached to the particle surface (e.g., poly(ethylene glycol), PEG or poly(vinyl pyrrolidone), PVP) [27,28] are often used to increase NPs stability in suspensions. The hydrophilic nature of these polymers also induces an extra stabilization through the short-range repulsive hydration forces [24]. Thus, steric stabilization results more adequate for biological systems. In contrast with the electrostatic stabilization, where the charge is defined during the synthesis steps of the NPs, steric stabilization usually requires an additional step of functionalization of the preformed colloids (Figure 1) [29,30]. Also, another major challenge in the use of colloids in biofluids is related to the tendency of certain compositional components (mainly proteins) of the fluid to unspecific coat the particles yielding the so-called “protein corona” [31,32,33,34,35]. This corona can cause two main issues: particle destabilization and surface inertization. These phenomena can decrease the circulatory lifetime of NPs in blood or inhibit their ability to bind the specific target receptors on cells and tissues [36].

Herein, we will revise the most common strategies used to achieve stable inorganic NPs in biological fluids and their interactions with proteins. Although special attention will be devoted to PEG coating of NPs, as the most widely used approach, other strategies using zwitterionic ligands, lipid bilayers, proteins, glycans, antibodies, aptamers, or amphiphilic polymers and/or the NPs immobilization on larger colloidal templates will be also discussed.

Overall, several established methods are available for imparting colloidal stability to inorganic NPs in biological media. However, they typically exploit surface ligands lacking of functional groups with targeting specificity. These functionalities are, in fact, commonly introduced a *posteriori*, in a second modification step. On the other hand, the used stabilizing ligands can significantly increment the hydrodynamic diameter of the NPs, thereby altering NPs properties such as mobility, blood circulation times, or cellular uptake. For instance, smaller hydrodynamic radii have much lower degrees of opsonization. Also, for efficient transmembrane permeation and excretion small hydrodynamic radius are required. Therefore, is obvious that size is a very important factor in the biodistribution of NPs [37]. Another nontrivial aspect in the selection of the stabilizing agents is the commercial availability, which is still restricted for several classes of ligands.

## 2. Surface Chemistry of Nanoparticles

**Figure 2:**
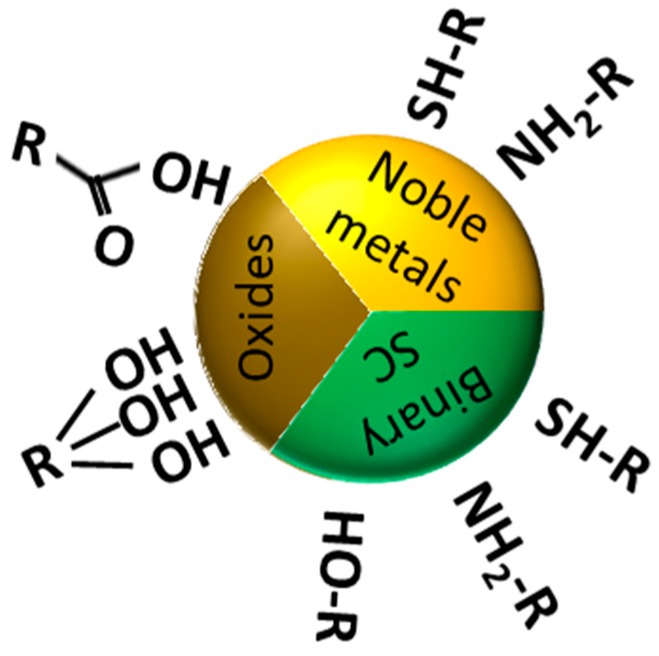
Schematic representation of the most commonly used functional groups in surface ligands and their preferred affinity towards binding different NP materials.

Regardless of the given ligand, a shared challenge is the incorporation of these molecules onto the NPs surfaces without precluding the colloidal stability during the functionalization process. This ligand exchange process has been widely studied and is highly dependent on the nanoparticle composition [18,38,39,40,41,42]. Therefore, depending of the NPs surface nature, different approaches have been developed based on the surface affinity towards different chemical groups. In general, we can recognize three main classes: (i) noble metals like Au and Ag (plasmonic materials) are normally functionalized with thiols or, to a lesser extent, amines and cyanides; (ii) oxides, typically used as components of magnetic NPs (e.g., iron oxides), can be easily coated via oxygen bonding with acidic and hydroxyl groups; (iii) binary compounds, particularly those including elements from Groups 12 to 16 as components of fluorescent semiconductor (SC) NPs (e.g., quantum dots), display high affinity towards thiols and hydroxyl groups, but also amino groups are often used. Figure 2 shows the most commonly used functional groups to coat NPs of different materials.

## 3. PEG as Stabilizing Agent in Biological Systems

To tackle the challenge of NP instability in biological fluids, many different approaches have been investigated [43,44]. Among them, those exploiting polymeric ligands have been shown to be effective because they provide the surface with a physical barrier preventing the NPs from coming into reciprocal contact. There are many suitable polymeric ligands to stabilize NPs in aqueous dispersions [45,46,47]. Nonetheless, when talking about aqueous solutions of high ionic strength, poly(ethylene glycol) (PEG) is by far the most widely used ligand to date [48,49] as it has the ability to provide the NPs with colloidal stability and biocompatibility in biological media [38,50,51,52,53,54,55]. One of the reasons for that is the highly hydrophilic nature of this polymer, providing steric stabilization and a short-range repulsive hydration layer around the particles that imparts an excellent long-term stability in high salt concentrations and extreme pH [56,57,58]. Another key factor regarding the preferential use of PEG is related to the presence of proteins in biological fluids. In fact, it has been demonstrated that PEG is capable of inducing a stealth effect of the NPs surface [59,60], which is reflected in the antifouling properties observed in PEG-coated NPs. Such a feature inhibits some non-specific interactions with proteins and, thus, delays the NPs opsonization process while reducing NP uptake in macrophage cells. This also leads to an improvement of the NP circulation time in blood by avoiding the reticuloendothelial system capture [37,61,62,63].

Besides all these advantages, PEG versatility also arises from the possibility to modify the PEG head groups a *la carte*, enabling not only the selective attachment onto NP surfaces, but also conferring multiple possibilities for further biofunctionalizations [58,64]. For instance, terminal functional moieties such as carboxylic (–COOH) and amine (–NH_2_) groups are widely use because can be introduced into PEG molecules without deteriorating the colloidal stability of the pegylated nanoparticles in blood and plasma [31]. Offering a valuable route for binding additional bioelements to impart specific properties to the NPs. Unfortunately, the successful coating of plasmonic NPs with PEG is highly dependent on the NPs material, surfactants, and surface stabilizers used in the NPs synthesis. Since these chemicals are required to control NP monodispersity, size, and shape, different approaches have been developed to replace the residual surface agents from NP surfaces with PEG after the synthesis.

### 3.1. PEG Coating of Plasmonic NPs

#### 3.1.1. Citrate-Stabilized Particles

**Figure 3:**
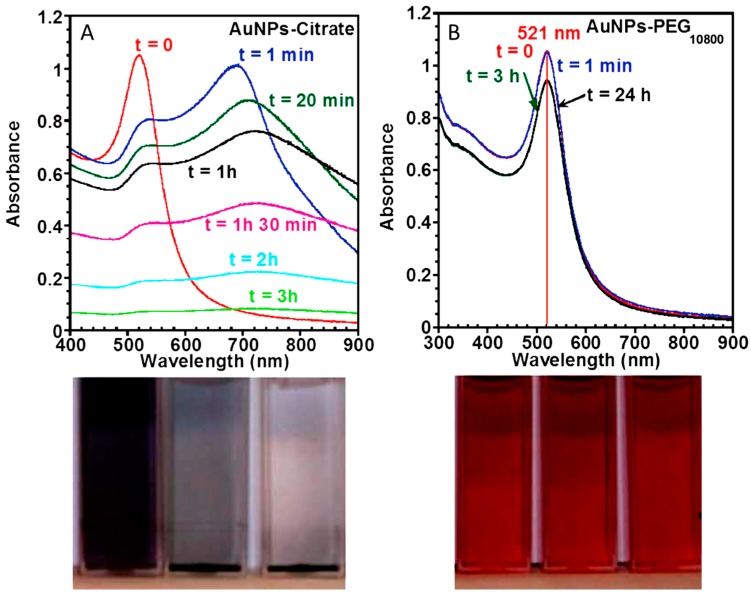
Colloidal stability of citrate-capped (**A**) and pegylated Au NPs (PEG_10800_) (**B**) in solutions with high salt concentration (0.157 M NaCl). The evolution of the colloidal state was monitored over time by UV–visible spectroscopy. The pictures of the corresponding colloidal solutions are shown in the middle, corroborating the high stability of the PEG–Au NPs colloidal solution under high ionic strength conditions, compared to Au NPs–citrate that aggregate and precipitate. Reproduced from [39] with permission.

**Figure 4:**
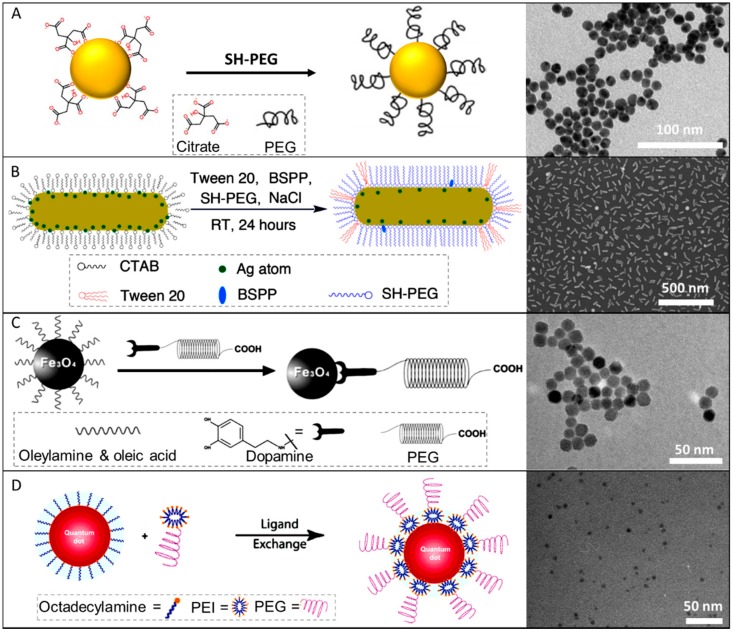
Schematic representation of different strategies for coating NPs with PEG. For plasmonic NPs, thiolated PEGs are by far the most widely used compounds in the pegylation process. A simple ligand exchange by adding SH-PEG when the colloids are citrate stabilized (**A**). If the surfactant is CTAB as for the case of Au NRs, the pegylation is conducted by successively adding surfactant Tween 20, bis(p-sulfonatophenyl) phenylphosphine dihydrate dipotassium (BSPP), HS-PEG, and NaCl into the CTAB-capped Au NRs solution, followed by its incubation at room temperature for 24 h (**B**). For the case of oxide particles as magnetite, the covalent attachment to the NPs surface is generally performed by oxygen bonding. In the presented example, dopamine was linked to carboxylic PEG to transfer the NPs into aqueous media and anchor PEG to their surface (**C**). For the case of QDs dispersed in organic phase, amines can be used for ligand exchange. In this case, octadecylamine was replaced with polyethylenimine (PEI) that was previously conjugated with PEG. In this way, PEG functionalization and the dispersion of QDs in water is achieved (**D**). Images reproduced from [40,41,66].with permission.

The most common and useful approach to coat plasmonic nanoparticles with PEG is the use of thiol-terminated derivatives (PEG-SH) that covalently graft onto the NP surfaces. For the case of citrate-stabilized NPs, this can be achieved by simply adding to the nanoparticles under stirring a solution of PEG-SH with the desired molecular weight. Over time, citrate ligands progressively exchange with PEG-SH, resulting in colloidal solutions that are very stable in high ionic strength solutions and biological fluids [39]. For instance, Holmes and co-workers [39] monitored the stability of the pegylated Au NPs under physiological conditions (0.157 M, NaCl) by UV–visible spectroscopy observing that the colloidal stability of PEG-stabilized NPs is only slightly affected upon the addition of a very high salt content. Figure 3 shows the time dependent spectra of citrate and PEG_10000_ stabilized Au NPs before and after addition of NaCl [39]. While unmodified citrate-stabilized Au NPs aggregate very fast, PEG stabilization prevents any significant alteration in the spectral profile. Moreover, the authors investigated the number of PEG-SH ligands required to fully coat the surface of Au NP of different diameters based on the given PEG molecular weight and NPs size. For instance, for 15 nm Au NPs, the maximum number of PEG molecules grafted to the NPs decreased with increasing PEG molecular weight. Specifically, the number of PEG molecules grafted to the Au nanoparticles decreased from 695 for PEG_2000_-SH to 50 for mPEG_51400_-SH. This indicates that the number of PEG molecules required to coat the surface decreases from 3.93 PEG nm^−2^ to 0.32 PEG nm^−2^ when increasing the PEG molecular weight from 2000 to 51,400 (see Table 1) [39,65]. On the other hand, Table 2 shows how the PEG grafting density varies depending on the particle size while Figure 4A outlines the coating procedure (a TEM image of the obtained PEG–Au NPs is also included).

#### 3.1.2. CTAB-Stabilized Au NPs

Unfortunately, only spherical-like nanoparticles can be produced using citrate, while different surfactants are necessary to produce anisotropic shapes. For instance, the synthesis of Au nanorods (NRs) is performed using cetyltrimethylammonium bromide (CTAB) as the surfactant. CTAB molecules are retained as a double-layer on the Au NRs after the synthesis. In this case, the ligand exchange process is quite delicate since particle aggregation or reshaping are quite common undesirable side events during the replacement procedure. In this context, Liao and Hafner [38] have developed a standard method to replace CTAB with PEG molecules on Au NRs via a one-step ligand exchange reaction using PEG-SH. Incorporation of tris-buffer at pH 3 during the process has shown to significantly increase the reaction kinetics [53]. However, this procedure was only able to displace the CTAB molecules at the tips of the NRs, and a large fraction of residual CTAB remained at the side surfaces [67]. Improvements in the pegylation efficiency were achieved by introducing a second functionalization step [52]. An alternative approach to promote the removal of residual CTAB profited from the compatibility of pegylated Au NRs with a diverse set of solvents (water, DMSO, THF, etc.) [68,69]. In this case, after replacing CTAB from the NRs tips with PEG-SH, ethanol is used desorb surfactant molecules from the NRs sides before carrying out a second pegylation step [52]. This method enables the complete functionalization of Au NRs with PEG even though a small quantity of CTAB was still observed on the NRs surface [52]. Complete surfactant removal was subsequently achieved devising a sophisticated strategy which initially reduced the CTAB concentration in the Au NR solution to ~1 mM (a threshold for which the Au NRs are still colloidally stable while the surface ligand exchange is more favorable) in the presence of Tween 20, a non-ionic stabilizing agent. After that, bis (psulfonatophenyl) phenylphosphine (BSPP), PEG-SH (Mw ≈ 2000 g/mol), and NaCl were successive added into the colloids. The mixture was incubated at room temperature for 24 h before submitting nanoparticles to washing cycles. Figure 4B shows a schematic representation of the surface functionalization of Au NRs with thiolated PEG and a representative TEM image [66]. We remark that pegylated Au NRs have been reported to be extremely stable in high ionic strength solutions, as for example 0.5 M NaCl and 20 mM phosphate buffer (pH 7.5) [66].

### 3.2. PEG Coating of Magnetic NPs

Sun and coworkers [40] have successfully anchored PEG on Fe_3_O_4_ nanoparticles through a covalent bond. Dopamine was first linked to one carboxylic group of a diacid PEG via the EDC/NHS chemistry. The so-derivatized PEG can then covalently anchor the surface of the particles thanks to the high affinity of dopamine to Fe_3_O_4_. Specifically, dopamine-modified PEG displaces the oleate and oleylamine residues used during the synthesis in a CHCl_3_/DMF solution to enable phase transfer to water. The stability of these particles was monitored in phosphate buffered saline (PBS) plus 10% fetal bovine serum (FBS) in a normal cell culture condition without observing any detectable agglomeration. A second effective approach to bind PEG onto oxide-based nanoparticles relies on silane-terminated PEG, as demonstrated by Zhang et al. [70] Figure 4C shows a schematic representation of the coating strategy with a TEM image of the obtained particles.

### 3.3. PEG Coating of Quantum Dots

Pegylation of quantum dots (QDs) is commonly performed via two main approaches depending on the specific synthetic approach (in organic vs. aqueous solvent). When QD are produced in organic solvents, a ligand exchange is also required to phase transfer the particles into water. This step can be performed simultaneously to the pegylation step. For instance, CdSe/CdS/ZnS QDs obtained in chloroform using octadecylamine (ODA) as a stabilizing agent can be transferred into buffer solutions in the presence of PEG-polyethylenimine [41]. The polyethylenimine moieties act as anchoring groups displacing ODA from the QD surfaces. Another approach to solubilize hydrophobic QDs exploits thiol-terminated molecules which firmly attach to the particle surface via the sulfur bond, providing solubility in aqueous media [71,72,73,74].

Alternatively, QDs has been directly produced in water using PEG as the stabilizing agent. For example, Scheper et al. [75] produced CdTe/CdS/ZnS QDs with a mixture of carboxyl and methoxy-PEG surface groups in aqueous solution. Furthermore, they also show that the QDs structural and emission stability in cell culture media was enhanced [75]. Figure 4D provides a schematic representation of the coating strategy with an image of the obtained pegylated QDs.

### 3.4. PEG Interaction with Proteins

**Figure 5:**
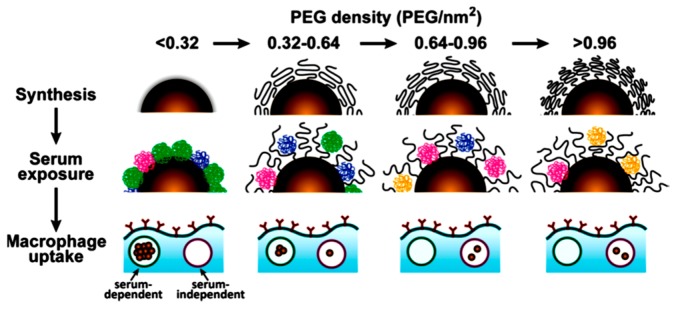
Schematic illustrating the influence of PEG density on surfaces serum protein adsorption to gold nanoparticles and their subsequent uptake by macrophages. The top panel shows gold nanoparticles grafted with PEG at increasing density. As PEG density increases, PEG volume decreases because of PEG−PEG steric interactions and a more compact layer is formed. The middle panel illustrates how PEG density determines the amount and relative abundance of serum proteins adsorbed to the gold nanoparticle surface after serum exposure. As the PEG density increases, fewer proteins are adsorbed. The lower panel shows that at low PEG densities, macrophage uptake is efficient and serum-dependent. At high PEG densities, macrophage uptake is driven predominantly by a less efficient serum-independent mechanism. Reproduced from [77] with permission.

As previously commented, when NPs are exposed to bio-fluids containing proteins, like human serum, a dense layer of proteins eventually adsorb on the NPs surface forming the so-called protein corona [76]. This has a tremendous impact on the NPs stability and their behavior in the biofluids. In fact, the protein corona plays a major role in determining the fate of NPs circulating in blood as well as their macrophage uptake, or even the blockage of their functionalities [31]. Thus, major efforts have been devoted to preventing or diminishing the formation of the protein corona. For instance, Chan and coworkers [77] showed that by increasing the number of PEG molecules (molecular weight of 5 kDa) on the NPs surface the total protein adsorption is nonlinearly reduced. The protein adsorption is predominantly a function of PEG grafting density for PEG structures with molecular weights above ~1 kDa [78,79]. At the highest PEG density tested (1.25 PEG/nm^2^), protein adsorption of serum was eliminated by 94–99% (relative to citrate-stabilized Au NPs). Importantly, the density of the PEG grafting, together with the NP size, also plays a key role in determining the specific composition of the protein corona, by reducing the adsorption of various proteins in serum to different extents. Moreover, it was reported that the PEG density also defines the mechanism and efficiency of the subsequent macrophage uptake. Below ~0.16 PEG/nm^2^, macrophage uptake depends on the presence of adsorbed serum proteins (serum-dependent uptake) meanwhile, above ~0.64 PEG/nm^2^, adsorbed proteins does not affect the macrophage uptake (serum independent uptake), see Figure 5 [77].

### 3.5. PEG Drawbacks

As it has been shown, pegylation of NPs, and especially using the thiolated derivate (PEG-SH), offers a convenient and reproducible post-synthetic functionalization method for imparting excellent colloidal stability in physiological media [80], and preventing undesired nanoparticle interactions with proteins or other blood components. However, PEG has also some drawbacks, such as immunogenic activity [55] or the considerable enlargement of the NP hydrodynamic diameters resulting from the necessity of using PEGs with a molar mass of at least around 5 kD (see Table 1 and Table 2). On the other hand, as previously discussed, even in the best case scenario of extremely high PEG densities, there is still a fraction of proteins that can adhere onto the NPs surfaces [77]. Furthermore, different PEG densities also influence the overall protein corona composition which has a direct impact on nanoparticle macrophage uptake. Additionally, Kiwada et al. [81] have demonstrated that, during in vivo experiments, the subjects can produce antibodies against PEG encapsulated nanocarriers, and therefore, subsequent doses of the same pegylated NPs can be rapidly cleared. PEG can also create unwanted immune responses. Moghimi et al. [82,83,84] have demonstrated the activation of complement systems by NPs functionalized with PEG, harnessing adverse immune responses and facilitating clearance by macrophages [85,86,87,88].

## 4. Zwitterionic Ligands

**Figure 6:**
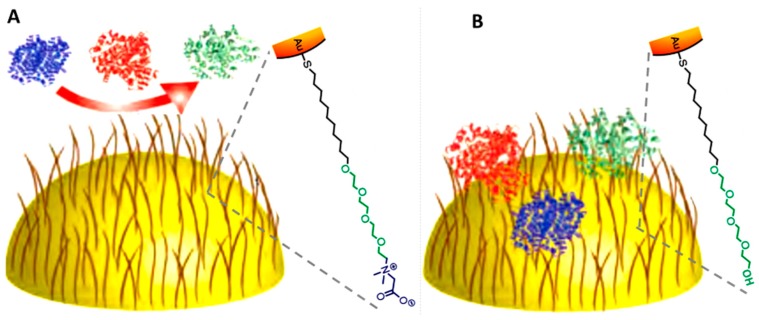
Illustration of the protein adsorption on the surface of a NP functionalized either with zwitterionic (**A**) or PEG (**B**) ligands. Both ligands have terminal thiol groups for binding to the Au surface. The zwitterionic ligand is composed of an oligo(ethylene glycol) chain followed by a carboxybetaine group (**A**). Reproduced from [85,94] with permission.

Zwitterionic ligands are molecules containing two or more functional groups with mixed positive and negative charges, and a net charge of zero. This makes these molecules lesser sensitive to high ionic strength solvents while also displaying similarities with proteins [89]. Consequently, they have been proposed as excellent ligands for NPs stability in biofluids. Notably, zwitterionic ligands have been reported to yield NPs with smaller hydrodynamic radii and much lower degrees of opsonization [37] than polymers like PEG [64], while also presenting remarkable stealthy properties [89]. In particular, zwitterionic ligands—including carboxybetaines and sulfobetaines—have been successfully used as anti-fouling coatings on NPs surfaces [90,91]. Carboxybetaines and sulfobetaines functionalities provide much lower adsorption of proteins and very low non-specific cellular uptake as well as increasing blood circulation times as compared to PEG coatings [92,93,94,95,96]. For instance, Jiang et al. [97] have demonstrated that poly (carboxybetaine) functionalized gold NPs were more stable in blood plasma and serum than PEG-coated NPs. Figure 6A shows a schematic representation of the chemical structure of a carboxybetaine zwitterionic ligand attached to a Au NP. Likewise, Mattoussi and coworkers [98] have synthesized zwitterionic QDs that showed great colloidal stability over a broad range of conditions including pH, salt, and undiluted serum. Using zwitterionic mixed monolayer on gold NPs, Rotello et al. [99] reported intracellular delivery of therapeutics while maintaining low uptake and minimal cytotoxicity from the nanomaterials. Mukherjee et al. [92] further demonstrated that zwitterionic gold NPs display higher blood circulation lifetime and enhanced tumor accumulation, while positively and negatively charged NPs were rapidly cleared. However, the surface charge distribution of zwitterionic NPs can influence the uptake and bio-distribution. Bawendi et al. [85,100] have reported that zwitterionic NPs exposing positive charges in their outermost layer show non-specific adsorption in vitro and in vivo, whereas zwitterionic NPs exposing negative charges in their outermost layer are far less susceptible to interactions with proteins. Figure 6B offers a comparison between protein adsorption on zwitterionic- or PEG-modified NPs.

### 4.1. Zwitterionic Coating of Plasmonic NPs

Zwitterionic plasmonic NPs are commonly fabricated using citrate-stabilized NPs and thiolated zwitterionic ligands. Typically, NPs are added dropwise to a solution containing these ligands and left under stirring for a certain period of time [94]. Another common approach is a ligand exchange in organic solvents to obtain aqueous stable dispersions. For instance, Rotello and coworkers [101] used first the common Brust–Schiffrin two-phase method in the presence of pentanethiol to produce Au NPs. This method consists in transferring tetrachloroaurate ions into toluene with tetraoctylammonium bromide for its subsequent reduction with sodium borohydride. The zwitterionic functionalization was subsequently performed via ligand exchange by mixing the so-formed Au NPs with the thiolated zwitterionic ligands in dichloromethane. After extensive washing, these NPs were redispersed in aqueous media such as PBS and cell culture media with high colloidal stability [101].

### 4.2. Zwitterionic Coating of QDs

Similarly as for plasmonic particles, surface functionalization of QDs with zwitterionic ligands is largely performed by using thiolated ligands [95,96,102]. As most of the synthetic schemes for QDs take place in organic solvents, a phase transfer step is typically required. For instance, Dubertret and coworkers [96] proposed a biphasic exchange of QDs dispersed in chloroform into an aqueous NaCl solution by direct mixing and stirring of the organic phase containing the QDs with an aqueous solution of dihydrolipoic acid sulfobetaine (DHLA-SB) as a zwitterionic ligand and phase transfer agent. Breus et al. [95] displaced the oleylamine surfactant from the QDs surfaces with the zwitterionic D-penicillamine (DPA) by refluxing the nanoparticles in 2-propanol and dioxane/methanol. On the other hand, when QDs are synthesized in aqueous solutions—mainly using biocompatible thiolated stabilizing agents [103,104,105,106]—an exchange procedure is not necessary. For instance, Kotov and Mamedova [107] produced CdTe QDs directly in aqueous media using the zwitterionic ligand l-cysteine as a stabilizer. However, this direct approach restricts the tuneability of the QDs and the exploitable zwitterionic molecules. Additionally, QDs synthesized in aqueous solutions usually have much broader size distributions which demand post-preparative size-selective precipitation procedures. Furthermore, these classes of QDs typically display a lower degree of crystallinity than the organometallic ones [103].

### 4.3. Zwitterionic Coating of Magnetic NPs

As the vast majority of magnetic NPs in biological applications are based on iron oxides, the production of water-soluble zwitterionic particles is usually achieved using ligands with functional moieties that provide strong coordination to oxide surfaces, like alcohol groups. Furthermore, since homogeneous magnetic NPs are preferably obtained via organometallic synthesis, a phase transfer step is also required. For instance, Bawendi and coworkers [108] produced iron oxide NPs from the thermal decomposition of Fe(CO)_5_ in dioctyl ether using oleic acid as ligand and trimethylamine N-oxide as additional oxidizing reagent to regulate the reduction kinetics. Water-soluble zwitterionic NPs were obtained by a two-step ligand exchange process. First, they exchanged the oleylamine by 2-[2-(2-methoxyethoxy)ethoxy]acetic acid in methanol to provide the NPs with solubility in a dimethylformamide/water mixture. Next, the particles were mixed with zwitterionic dopamine sulfonate at 70 °C for 12 h. After cleaning, the NPs were dispersed in PBS at pH = 7.4 [108].

### 4.4. Drawbacks of Zwitterionic Coatings

Unfortunately, most zwitterionic ligands used in literature are not commercially available and require complex synthetic protocols. Additionally, another inconvenience of this type of ligands is that, under slight stimulus such as minor changes in the pH, their behavior can alternate from being unseen for the proteins (stealthy) to inducing their adsorption on the NPs surface (sticky) [109].

## 5. Lipid Bilayer

Another strategy to impart stability in biological media and produce a stealth effect on NPs relies on the use of lipid bilayers as coating agents. The rationale of this approach is to mimic the exoplasmic leaflet of cell membranes for gaining biostability [110]. In contrast to polymers and other synthetic molecules, which often produce heterogeneous coatings, lipid bilayers typically create thin homogeneous shells around the NPs with an average thickness of 5 nm. Therefore, the hydrodynamic diameter of the NPs does not undergo of dramatic changes after the coating [111]. Also, as previously highlighted, the high homogeneity of the coatings is a key factor to avoid protein adsorption. The presence of residual areas of uncoated NP surfaces is the reason why the expected stabilizing effect of several polymers is not always obtained, and its high dependency on factors such as polymer chain length and the conformation of the chains at the NPs surface [112]. Differently, it has been reported that the coating of Au NPs with a lipid bilayer of 1,2-dimyristoyl-sn-glycero-3-phosphocholine (DMPC) is extremely homogeneous, therefore providing large stability in a suspension of HBS 150 mM NaCl, pH 7.4 and in cell culture medium (RPMI), containing or not SVF 10% proteins [88]. Furthermore, Bhowmik et al. [113] used a lipid bilayer composed of POPC (1-palmitoyl-2-oleoyl-sn-glycero-3-phosphocholine), POPG (1-palmitoyl-2-oleoyl-sn-glycero-3 phosphoglycerol), and cholesterol to mimic a cell membrane. The coated NPs suspension showed high stability in a high salt containing (100 mM, NaCl) solution and also in phosphate buffer [113].

### 5.1. Lipid Bilayer Coating of Plasmonic NPs

**Figure 7:**
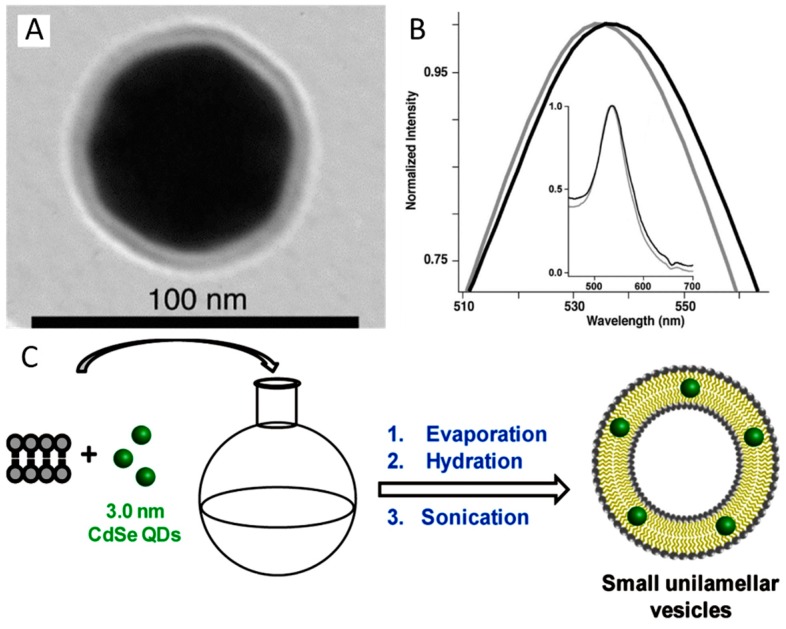
(**A**) TEM image where it can be clearly seen a lipid bilayer (dioleoylphosphatidylcholine, DOPC), egg sphingomyelin (ESM), and ovine cholesterol (Chol)) on a Au NP. (**B**) Normalized UV–vis absorption spectra of citrate (grey) and lipid-coated (black) Au NPs. The LSPR peaks redshift from 534 nm to 538 nm after the lipid coating. (**C**) Formation of L-QD vesicles via solvent evaporation, hydration and probe sonication. Reproduced from [114,115] with permission.

Typically, coating of plasmonic NPs in aqueous suspensions with lipid bilayers requires first the formation of a lipid film. To this end, lipids such as dioleoylphosphatidylcholine (DOPC), egg sphingomyelin (ESM), and ovine cholesterol (Chol) are dissolved together in a mixture of chloroform/methanol. Next, the solution is dried under a stream of argon and vacuum to remove any residual solvent. For the NPs encapsulation, the films are previously hydrated with water and then plasmonic particles are added and sonicated to produce unilamellar vesicles [114]. Figure 7 shows a single Au NP coated with a lipid bilayer and the corresponding UV–vis spectra of the colloidal suspension before and after coating [113,114]. The redshift of the localized surface plasmon resonance (LSPR) is ascribed to the increasing of the refractive index upon coating with the lipid bilayer.

### 5.2. Lipid Bilayer Coating of Fluorescent NPs

Typically, chloroform solutions of lipid and QDs (0.5 nmol) are mixed and then dried. The dried film is subsequently hydrated with water. During this step, multilamellar vesicles containing QDs are obtained. Further sonication of this dispersion induces the conversion of the multilamellar vesicles into small unilamellar ones containing QDs [115]. In contrast to plasmonic and magnetic particles, the obtained lipid bilayers typically contain several QDs because of the small NP size.

### 5.3. Lipid Bilayer Coating of Magnetic NPs

The lipid bilayer wrapping of iron oxide is usually achieved by mixing a suspension of DMPC with the NPs. The NPs induce first the rupture of the DMPC vesicles and then the NP wrapping. Further sonication can obtain small unilamellar vesicles. Such DMPC-coated magnetic NPs have shown stability in a high ionic strength medium (Hepes 20 mM, NaCl 150 mM, pH = 7.4) [116].

### 5.4. Drawbacks of Lipidic Coatings

Lipid coatings have been shown to provide NPs with stability over several days in very different solutions including the most common media used in cell culture. However, it has also been reported that the NP aggregation with lipid coatings in some culture media with high concentrations of cysteine or glutathione [117]. Importantly, one of the main drawbacks of using lipid bilayers is the lack of covalent binding onto the NP surfaces, which can lead to lipid detachment and, thus, particle aggregation.

## 6. Protein Coatings

When NPs are dispersed in fluids containing proteins, the formation of the protein corona typically causes NPs destabilization (and agglomeration) and passivation of the surface functional elements [118,119,120,121,122]. However, if such a process is properly controlled, it can be exploited in an advantageous way. In fact, it is known that proteins are macromolecules with good stability under physiological conditions and with numerous charged groups that can provide NPs with steric and electrostatic stabilization. Moreover, the ability of proteins to generate a hydration shell via hydrogen bonds with water molecules prevents the adhesion of other proteins [123]. Consequently, an appropriately pre-protein coating on the NPs surface before its addition to a biofluid can provide colloidal stability in physiological media meanwhile avoiding at the same time the surface attachment of other unwanted free proteins in the media. Among different proteins, serum albumin is particularly suited to this use since it carries cysteine groups which are highly reactive toward metallic surfaces [119,124]. On the other hand, serum albumin is one of the main proteins forming the protein corona [125]. Additionally, the electrostatic interactions between amino acids and NPs (e.g., positively-charged amino acids such as lysine, arginine, and histidine; and negatively-charged aspartic and the glutamic acids residues) plays an important role in the surface adsorption.

### 6.1. Protein Coating of Plasmonic NPs

NP coatings with proteins can be easily produced by ligand exchange. In the case of citrate-coated gold NPs, the functionalization procedure is rather straightforward. Usually, a protein/citrate solution with pH of 7–8 is added dropwise to the colloidal suspension under vigorous stirring. On the other hand, for different surface agents, the protein coating can be more tedious. For instance, in the case of CTAB-capped Au NRs, the surfactant concentration has to be first lowered to the critical micelle concentration (CMC = 1 mM). Then, the NRs dispersion is added drop by drop to a bovine serum album (BSA)/citrate solution at pH 7 under sonication. NRs are further centrifuged and resuspended again in BSA/citrate solution at pH 12. Finally, extensive NP washing is performed with water at basic pH (pH 11–12) [126]. For instance, Kreyling and coworkers [127] coated Au NPs with human serum albumin (HSA) and apolipoprotein E (apoE) which is also frequently found in the protein corona formed from blood and serum [128,129]. On the other hand, Parak and coworkers [130] coated citrate Au NPs with various proteins of different molecular weights (insulin (5808 Da, Ins), b-lactoglobulin (18.3 kDa, monomeric, b-LG), BSA (64 kDa), ovalbumin (45 kDa, Ova-DQ), rhodamine-labeled insulin (Ins-R), and rhodamine-labeled bovine serum albumin (BSA-R)) [130,131,132]. All these protein-encapsulated NPs exhibited remarkably high colloidal stability in physiological media, including various culture media containing salts and proteins. Furthermore, they display bio-degradability upon enzymatic digestion, which might be exploited for drug-delivery applications [130,133].

### 6.2. Protein Coating of QDs

**Figure 8:**
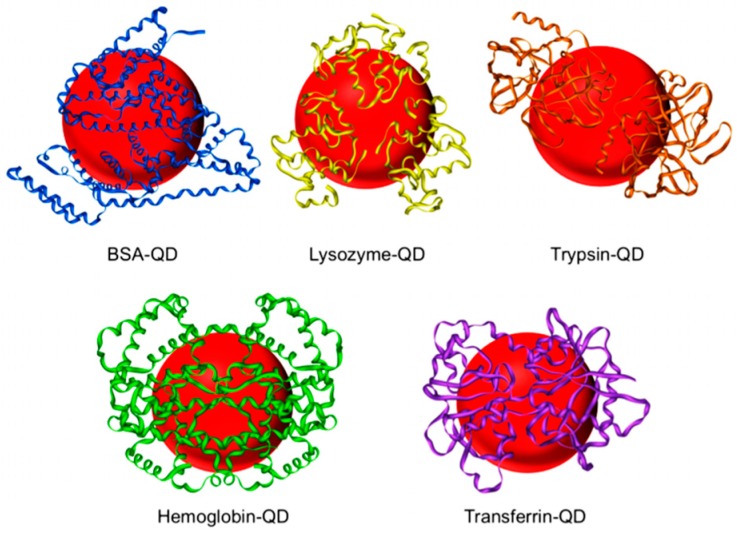
Schematic illustration of BSA-, lysozyme-, trypsin-, hemoglobin-, and transferrin-functionalized QDs. Reproduced from [134] with permission.

Xuewen and Ma [134] reported the one-step synthesis of protein-functionalized QDs. Zn(OAc)_2_ and Hg(ClO_4_)_2_ were mixed with a protein solution in PBS (e.g., BSA, lysozyme, trypsin, hemoglobin, and transferrin) in the presence of mercaptopropionic acid and NH_4_HCO_3_. Then, NaHSe was quickly injected into this solution inducing the formation of Zn_x_Hg_1−x_Se QDs. Figure 8 shows a schematic representation of different proteins coating on QDs [134].

### 6.3. Protein Coating of Magnetic NPs

Rosenfeld and coworkers [135] coated magnetic NPs with BSA, HSA, and thrombin (TR). The production of magnetite was done by the co-precipitation of ferrous and ferric salts in water. For protein coating, the magnetic NPs were added to the different protein solutions in phosphate buffer at pH 6.5 (BSA, HSA) or pH 7.3 (TR) in the presence of hydrogen peroxide solution.

### 6.4. Drawbacks of Protein Coatings

As for other ligands, one of the main issue associated with protein coating of NPs is related to the thickness of the coating layer that considerably increases the hydrodynamic diameter of the NPs. Moreover, since protein solutions are generally obtained in PBS buffer solutions to avoid their denaturation, the high ionic concentration can pose problems to NPs stability at the beginning of the coating process.

## 7. Glycans

Glycans are important biomolecules that can selectively bind to clinically relevant proteins and therefore are essential components in inter- and intracellular signaling processes [136,137]. Biocompatible, non-plasmonic gold nanoparticles (AuNPs, size < 3 nm) coated with thiol-terminated glycans (glyconanoparticles) have been widely reported, and their use mainly focused toward exploiting the multivalent features of carbohydrates to address different biological problems [138]. Notably, the selectivity of glycans toward specific cell types, through targeting carbohydrate-binding receptors, is maintained even in protein-rich media [138]. Therefore, functionalization of NPs with glycans not only ensures colloidal stability in protein-rich physiological media but also prevents phagocytosis by macrophages and exhibits excellent selectivity toward carbohydrate binding proteins (lectins).

### 7.1. Glycans Coating of Noble NPs

**Figure 9:**
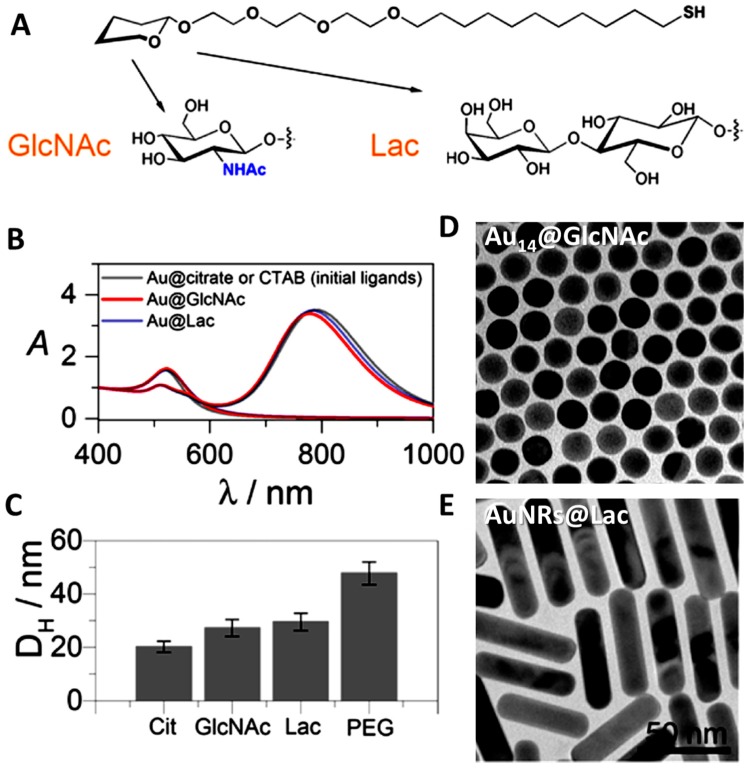
Molecular structures of thiol-terminated neoglycoconjugates of *N*-acetylglucosamine (GlcNAc) and lactose (Lac) (**A**). UV−vis−NIR spectra of Au nanospheres and NRs stabilized with their corresponding glycan and original ligands (**B**). Hydrodynamic diameters measured in water of Au NPs stabilized with different ligands (**C**). TEM images of Au nanospheres and NRs stabilized with GlcNA cand Lac (**D**,**E**). Reproduced from [139] with permission.

Liz-Marzan and coworkers [139] functionalized Au nanorods and citrate-stabilized nanospheres using thiol-terminated glycoconjugates either N-acetylglucosamine (GlcNAc) or disaccharide lactose (Lac) (Figure 9A,B shows their molecular structures, and their corresponding UV−vis−NIR spectra before and after functionalization). These particles incubated in a standard cell culture medium (Dulbecco’s modified Eagle’s medium, DMEM) supplemented with 10% fetal bovine serum (FBS) show no changes in the intensity or in the position of their LSPR bands. Moreover, the hydrodynamic diameter (DH) of Au protected with these glycans was found to increase from 20 nm for citrate-stabilized particles, up to 27 and 30 nm for GlcNAc and Lac coatings, respectively. This size increment is quite low if compared with the same NPs stabilized with 5 kDa PEG (DH of 49 nm, Figure 9C). Figure 9D,E displays representative TEM images of particles after glycans functionalization [139]). The coating process is generally quite simple; a thiol-terminated glycan is directly added dropwise to the NPs solution under vigorous stirring and allowed to react. It has been reported that this approach provides colloidal stability in biological fluids, prevents the formation of the protein corona, and maintains targeting functionality in protein-rich physiological media.

### 7.2. Glycans Coating of QDs

Seeberger’s group prepared CdTe@ZnS nanoparticles with different emission maxima in organic solvents obtaining pyridine coated QDs. Surface-modification with carbohydrates was achieved by mixing a solution of the QDs in dichloroethane and a solution of 2-(2-(2-thioethoxy)ethoxy)ethoxy-b-d-galactopyranoside in dichloroethane/ethanol at 50 °C. The sugar-coated QDs were purified by precipitation from a mixture of n-hexane/chloroform/methanol and subsequently dissolved in water [140].

### 7.3. Glycans Coating of Magnetic NPs

**Figure 10:**
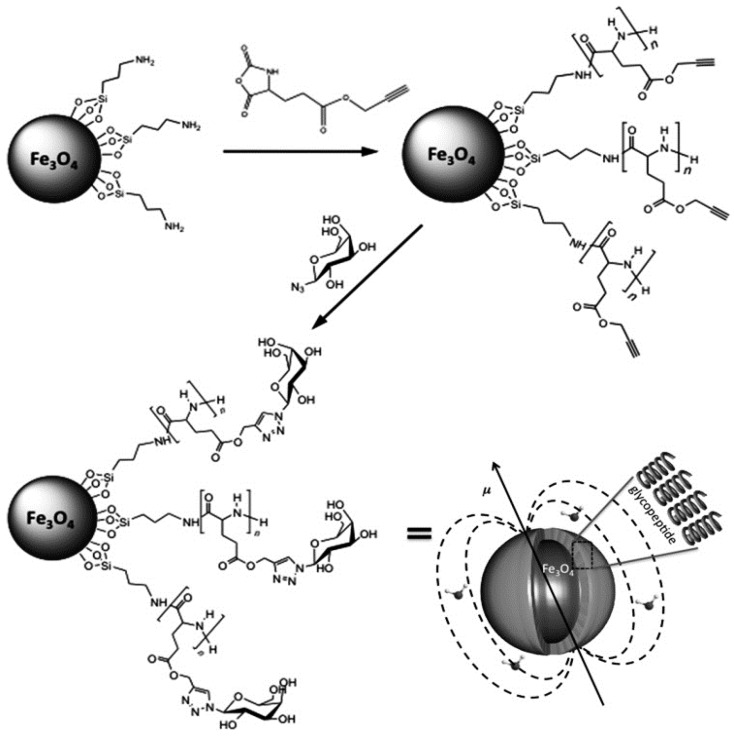
Schematic representation for the synthesis of glycopeptide-grafted magnetic nanoparticles. Reproduced from [142] with permission.

The synthetic procedure for coating magnetic NPs with sugars (Figure 10) involves the preparation of Fe_3_O_4_ using a reported surfactant-free method [141]. The NPs were further functionalized with 3-aminopropyl-triethoxysilane (APTS). After that, the surface-amines initiate the ring-opening polymerization of propargyl-l-glutamate, producing polypeptide grafted NPs with clickable alkyne groups. Glycosylation was achieved by the Huisgen click reaction of azide-functionalized galactose to the surface. The resulting glycol peptide grafted particles were easily dispersible in water saline buffer and in serum [142].

### 7.4. Drawbacks of Glycans Coatings

Unfortunately, thiol glycoconjugated sugars for Au NPs are not commercially available while complicated chemistry procedures have to be performed on the NPs’ surface especially for magnetic and QDs.

## 8. Poly(Maleic Anhydride) Based Polymers

**Figure 11:**
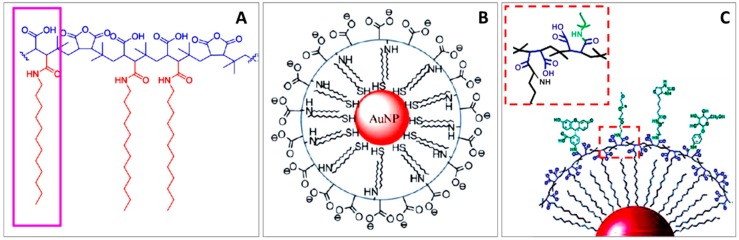
Structure of the amphiphilic polymer poly (isobutylene-alt-maleic anhydride) (PMA), functionalized with dodecylamine. The purple box shows a monomer unit. The hydrophobic and hydrophilic parts are drawn in red and blue, respectively (**A**). Scheme of a Au NP transferred from chloroform to water using PMA-dodecylamine polymer (**B**). Incorporation of additional functionalities in the polymer around the particles linked thought an amino-terminal group to the polymer and lose look to the amino group linkage to the polymer (**C**). Reproduced from [63,143] with permission.

These type of coatings are based on the use of amphiphilic polymers that are constructed using poly(maleic anhydride) (PMA) as the hydrophilic backbone. The use of alkylamines to form hydrophobic side chains by reacting some of the anhydride rings of the PMA with the amino groups allows the polymer to intercalate with the hydrophobic surfactant layer on the NPs surface. Then, by linking some of the remaining anhydride rings with diamine linkers, a cross-linked shell is formed by the polymer molecules around each nanoparticle. As a result, particles can be transferred to an aqueous solution. In this process, the remaining anhydride rings from the PMA backbone open and yield negatively charged carboxyl groups, providing stability to the NPs. These carboxylic groups can be further used to covalently link different functional molecules to the NPs surface (see Figure 11) [63,143]. For instance, molecules such as PEG could be easily attached to the particle surface via 1-etil-3-(3-dimetilaminopropil)carbodiimide (EDC) chemistry. Importantly, the authors showed that the polymer could be directly modified with biomolecules, before the NPs coating step, and therefore avoiding eventual colloidal instabilities during the linkage process. Moreover, besides PEG, additional functionalities can be incorporated in the polymer around the particles to introduce specific binding sites (e.g., biotin, galactose), or fluorescence (e.g., fluorescein) [63,143,144]. Parak and coworkers [143] developed this approach for coating hydrophobic nanoparticles and it to aqueous solution. They also demonstrated its applicability regardless of the NP material (plasmonic, fluorescent, or magnetic NPs). For instance, this approach has been successfully used with Au, CdSe/ZnS, and Fe_3_O_4_ among others. The obtained NPs remain stable under biological conditions without aggregation [145].

### 8.1. Poly(Maleic Anhydride) Based Polymers Coating of Plasmonic, Fluorescent and Magnetic NPs

In general, polymer(isobutylene-altmaleic anhydride) is vigorously mixed with dodecylamine in THF. Upon heating for several hours, the amine groups react with the anhydride rings. Next, a solution of the polymer in chloroform and a solution of hydrophobic monodispersed nanocrystals in chloroform are mixed and stirred at room temperature. After evaporation of the solvent, bis(6-aminohexyl) amine in chloroform was then added to cross-link the polymer shell that had formed around each nanocrystal [143,146,147].

### 8.2. Drawbacks of Glycans Coatings

A significant drawback with this technique is that the large molecular weights of the polymers substantially increase the NP hydrodynamic diameter. Furthermore, this procedure requires a very homogenous NP system since a key point in this approach is the correct estimation of the number of monomer units that needs to be added per nm^2^ of effective NP surface area. This calculation is also crucial to further use only some of the anhydride rings of the PMA with the amino groups of the alkylamines and the rest anhydride rings of the PMA to cross-link the shell. Additionally, the polymer coating should be carried out in diluted NP suspension (NP concentration ≤ 1 μM).

## 9. Mercaptoalkyl Acid Ligands

Among the different mercaptoalkyl acid ligands, 11-mercaptoundecanoic acid (MUA) is one of the most commonly used in surface functionalization of NPs. MUA itself provides less stability to the NPs as compared to, for instance, PEG. However, it has been shown that the role of MUA ligands can be enough to protect NPs with a rather thin monolayer, while the carboxylic group of MUA can be exploited for subsequent bio-conjugations (e.g., via EDC protocols). In this way, colloidal stability is provided to the NPs in biological fluids, like serum and blood, while simultaneously introducing extremely effective targeting and multiplex capabilities for bio-detection in real biological fluids (e.g., bacteria identification) [18].

### 9.1. Mercaptoalkyl Acid Ligands on Plasmonic NPs

Generally, citrate-stabilized NPs are selected as plasmonic NPs and functionalized with mercaptoalkyl acid ligands by direct addition. For instance, Ag NPs were functionalized with MUA by rapidly adding a MUA/ammonia solution (basic pH) to Ag NPs dispersion under vigorous stirring. The obtained MUA functionalized NPs were further conjugated with different antibodies via carbodiimide chemistry. Obtained NPs not only were stable in PBS solutions during Ab conjugation, but they were also extremely stable in serum and real blood samples after conjugation [18,148].

### 9.2. Mercaptoalkyl Acid Ligands on QDs

An easy and frequently-used method to prepare water-soluble QDs is to synthesize the NPs in organic solvents and then perform a phase transfer by replacing the original surfactants with mercaptoalkyl acid ligands [149,150,151]. The thiol moiety binds to the QD surface and replaces ligands such as TOPO and oleylamine. Upon deprotonation, the carboxylate group of the ligand grants water solubility to the QD. In general, a certain number of a mercaptoalkyl acid ligand like MUA is mixed with the QDs in methanol at basic pH. After providing energy to the system as heat, the ligand exchange takes place and the QDs become hydrophilic [152]. These types of ligands have been shown to provide stability to the QDs in phosphate buffer (50 mM, pH 7.2) solutions [153].

### 9.3. Mercaptoalkyl Acid Ligands on Magnetic NPs

When magnetic nanoparticles based on Fe, Ni, or Co oxides are employed, surface ligands with functionalities such as ethoxy or carboxylates groups are used instead of the thiolated ones. However, although less frequently used, magnetic NPs can be produced with materials displaying affinity towards thiols. For example, FePt nanoparticles produced with oleic acid were transferred to water using MUA. The FePt NPs were dispersed in a hexane/octane mixture and a MUA solution in cyclohexanone was added. After several washing cycles, the NPs were finally dispersed in basified aqueous solution [154]. Alternatively, prior coating of magnetic particles with a layer of Au or Ag offers also a way to subsequently anchor thiolated molecules onto the NP surfaces [155].

### 9.4. Drawbacks of Mercaptoalkyl Acid Ligands

Molecules like MUA are usually short providing low increase in the hydrodynamic radii but to the detriment of colloidal stability as compared to molecules like PEG. Also, such colloidal stability is highly pH dependent. On the other hand, a reduction in QD quantum yield and chemical stability has been reported for mercaptoalkyl acids ligand [152]. Finally, these ligands display poor binding affinity towards the most common magnetic particles.

## 10. Aptamers

While several functionalization strategies are capable of providing colloidal stability in biological media, they largely fail to impart any chemical specificity and active targeting elements are typically introduced a *posteriori* [18,122,156,157,158]. Multiple efforts have been devoted to address this challenge with the attempt to simultaneously afford particle stability and biorecognition functions. In this field, aptamers emerged as valuable alternatives to more traditional biorecognition elements such as antibodies. Firstly, aptamers combine the excellent targeting specificity of Abs while providing key improvements in terms of stability, ease of production, small size, and immunogenicity. Secondly, aptamer-NPs can be obtained in a one-step process. Thus, aptamers are emerging as the new generation of recognition elements in nanobio-applications.

### 10.1. Aptamer Coating of Plasmonic NPs

**Figure 12:**
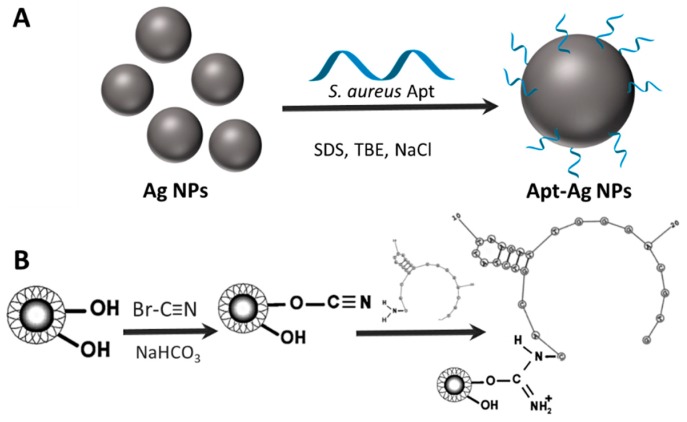
Schematic representation for the bioconjugation of citrate-stabilized Ag NPs with a thiolated *S. aureus* aptamer (**A**) and starch-coated magnetic NPs with amino-modified E1E2-6 aptamer (**B**). Reproduced from [159,163] with permission.

Thiol-modified aptamers can be directly assembled onto the surface of plasmonic NPs through thiol bonding. For instance, a 5′-alkyl-thiol-modified *S. aureus* aptamer was self-assembled onto the surface of Ag NPs by direct addition to the colloidal suspension in the presence of sodium dodecyl sulfate, TBE buffer, and sodium chloride (see Figure 12A). In this case, no extra stabilizing agents were necessary as the aptamer surface modification maintains the colloidal integrity of the particles. The so-modified particles have been shown to be stable in serum, and in several real human fluids—such urine, blood, or pleural effusion and ascites—while retaining their biorecognition capabilities [159].

### 10.2. Aptamer Coating of QDs

Thiol-modified aptamers can be also used to functionalize QDs. For example, the thiolated aptamers were covalently coupled to the CdSe/ZnS QDs surface to detect thrombin and cocaine [160,161]. In general, QDs stabilized in organic solvents are firstly transferred to water prior to the aptamer conjugation. For instance, Zhou and coworkers [160] used first 3-mercaptopropionic acid (MPA) in a mixture chloroform/methanol to replace the QDs ligand (TOPO) with a thiol at basic pH to deprotonate the carboxylic groups of MPA and obtain water-soluble QDs. After the QDs were transferred to water, they were dispersed in a solution containing the thiol-ended aptamer and aged for 12 h [160].

### 10.3. Aptamer Coating of Magnetic NPs

The most common approach to functionalize magnetic nanoparticles with aptamers exploits terminal amino groups of the ligands to be coupled to hydroxy or carboxyl functionalized NPs via EDC chemistry. Aptamer functionalized magnetic NPs have been shown to be stable in PBS solutions [162,163]. Delavid et al. [163] activated the hydroxyl groups of starch-functionalized magnetic NPs (using cyanogen bromide in a sodium bicarbonate buffer) to couple them with amino groups of aptamers (Figure 12B). On the other hand, Kumar group [162] used a similar approach anchoring via EDC chemistry amino-modified aptamers onto carboxylated dextran-stabilized magnetic NPs.

### 10.4. Drawbacks of Aptamer Coatings

Currently, the main problem related with this class of ligands is the relatively reduced library of commercially available aptamers.

## 11. NPs Immobilization on Colloidal Substrates

Another strategy that can maintain NP properties in biological fluids while avoiding aggregation relies on their immobilization on larger colloidal templates, such as silica or polymeric microbeads. For instance, Ag nanoparticles have been electrostatically accumulated on silica microbeads colloidally stable in biofluids, such as blood. Plasmonic surfaces were then functionalized with a specific peptidic chemoreceptor for the oncoproteins. Quantification of the target biomolecule was then obtained in real blood samples via surface-enhanced Raman scattering (SERS) assay [164]. Another example of such hybrid systems is silver-coated aluminum microrods [165]. Here, porous Al microrods (2 µm) produced by simple sonication were coated with small Ag nanoparticles which, in a subsequent step, were further overgrown to a desired size. The produced system which showed very good stability in PBS [165].

## 12. Conclusions

In conclusion, there are several molecules that can be used to preserve the integrity and stability of NPs in biological fluids like PEG, zwitterionic ligands, glycans, aptamers, etc. The strategies to anchor these ligands onto the NPs surfaces are quite diverse and highly dependent on the NP composition reflecting the different binding affinity towards a specific material. In any case, we can recognize three main classes of functional groups (thiols, amines, and hydroxyls) that are generally used to anchor ligands to the NPs surfaces. In general, thiols are used to covalently bind molecules on the surface of gold and quantum dots. For the case of oxide particles like iron oxide, molecules with hydroxyl terminations are used to promote oxygen bonding. Table 3 shows a summary of the most common approaches used to functionalize the surfaces of nanoparticles. On the other hand, many of the ligands used are not commercially available, requiring complex synthetic routes and therefore considerably restricting their applications and the investigation on their behavior in biological media.

## Figures and Tables

**Table 1:** Surface coverage (from TGA) and PEG-SH layer thickness (from DLS size distribution by volume) on 15 nm gold nanoparticles. Adapted from [65] with permission.

PEG-SH (*M*_w_)	DLS (v)/PEG Layer (nm)	Weight Loss (%) T > 320 °C	N_PEG_ per 15 nm AuNP	Footprint (nm^2^)	Grafting Density (nm^2^)
2100	2.83 ± 0.66	6.7	695 ± 87	0.25	3.93
5400	7.79 ± 1.0	9.9	424 ± 53	0.42	2.4
10,800	12.77 ± 1.5	12	278 ± 42	0.63	1.57
19,500	21.61 ± 2.5	10.82	123 ± 16.5	1.33	0.75
29,500	25.6 ± 3.0	10	81 ± 10	2.18	0.46
51,400	37.15 ± 4.0	10.85	50 ± 6	3.15	0.32

**Table 2:** Surface coverage (from TGA) and PEG-SH layer thickness (from DLS size distribution by volume) on different gold nanoparticles sizes coated with PEG_10000_. Adapted from [65] with permission.

Diameter (nm)/EM	Diameter (nm)/DLS	Weight Loss (%) T > 320 °C	N_PEG_ per AuNP	Footprint (nm^2^)	Grafting Density (nm^2^)
15 ± 1.8	59 ± 3.5	14.25	278 ± 42	0.63	1.57
30 ± 3.5	72 ± 5	5.7	916 ± 106	0.78	1.29
62.5 ± 6	102 ± 9	1.64	2572 ± 402	1.25	0.8
93 ± 12	138 ± 10	1.41	6778 ± 814	1.05	0.96
115 ± 10	165 ± 14	1.45	12,960 ± 1227	0.8	1.25

**Table 3:** Summary of the most common reported approaches.

	Material		
Stabilizing Molecule	Plasmonic Particles	Magnetic Particles	Quantum Dots
PEG	SH-PEG	Hydroxyl-PEG (dopamine-PEG)	PEI-PEG
			SH-PEG
Zwitterionic ligands	SH-zwitterionic	Dopamine-zwitterionic	SH-zwitterionic
Lipid bilayers	DOPC/ESM/Chol	DMPC	POPC/POPG
Protein coatings	Serum Albumin Insulin Lactoglobulin Ovalbumin	Serum Albumin Thrombin	Serum Albumin Lysozyme Trypsin Hemoglobin Transferrin
Glycans	SH-glycoconjugates	Azide-Galactose	Thioethoxy-galactopyranoside
Poly(maleic anhydride) based polymers	polymer(isobutylene-altmaleic anhydride)/dodecylamine/bis(6-aminohexyl)amine		
Mercaptoalkyl acid ligands	MUA		
Aptamers	SH-Apt/SDS/TBE/NaCl	NH_2_-Apt	SH-Apt
